# Photosynthesis and Related Physiological Parameters Differences Affected the Isoprene Emission Rate among 10 Typical Tree Species in Subtropical Metropolises

**DOI:** 10.3390/ijerph18030954

**Published:** 2021-01-22

**Authors:** Junyao Lyu, Feng Xiong, Ningxiao Sun, Yiheng Li, Chunjiang Liu, Shan Yin

**Affiliations:** 1 School of Agriculture and Biology, Shanghai Jiao Tong University, Shanghai 200240, China; lvjunyao@sjtu.edu.cn (J.L.); xfsgsb@sjtu.edu.cn (F.X.); nxsun@sjtu.edu.cn (N.S.); yyhhli@stanford.edu (Y.L.); chjliu@sjtu.edu.cn (C.L.); 2 Shanghai Urban Forest Ecosystem Research Station, National Forestry and Grassland Administration, Shanghai 200240, China; 3 Yangtze River Delta Ecology & Environmental Change and Control Research Station, Ministry of Education, Shanghai 200240, China; 4 Department of Biomedical Data Science, Stanford University School of Medicine, Stanford, CA 94305, USA; 5 Key Laboratory for Urban Agriculture, Ministry of Agriculture and Rural Affairs, Shanghai 200240, China

**Keywords:** BVOCs, TOF-MS, photosynthesis, isoprene, tree species

## Abstract

Volatile organic compound (VOCs) emission is an important cause of photochemical smog and particulate pollution in urban areas, and urban vegetation has been presented as an important source. Different tree species have different emission levels, so adjusting greening species collocation is an effective way to control biogenic VOC pollution. However, there is a lack of measurements of tree species emission in subtropical metropolises, and the factors influencing the species-specific differences need to be further clarified. This study applied an in situ method to investigate the isoprene emission rates of 10 typical tree species in subtropical metropolises. Photosynthesis and related parameters including photosynthetic rate, intercellular CO_2_ concentration, stomatal conductance, and transpiration rate, which can influence the emission rate of a single species, were also measured. Results showed *Salix babylonica* always exhibited a high emission level, whereas *Elaeocarpus decipiens* and *Ligustrum lucidum* maintained a low level throughout the year. Differences in photosynthetic rate and stomatal CO_2_ conductance are the key parameters related to isoprene emission among different plants. Through the establishment of emission inventory and determination of key photosynthetic parameters, the results provide a reference for the selection of urban greening species, as well as seasonal pollution control, and help to alleviate VOC pollution caused by urban forests.

## 1. Introduction

Urban forests are a vital part of the urban ecosystem. Research on the relationship between urban forests and atmospheric pollutants has become a popular topic in urban ecology [1,2,3]. Biogenic volatile organic compounds (BVOCs) are a special class of plant metabolites [4]. Most plants produce BVOCs and it is estimated that there are more than 30,000 species [5].

BVOCs are the main source of volatile organic compounds (VOCs) globally [6]. It is estimated that the average annual emission of VOCs is 1273 TgC, and 1150 TgC is derived from BVOCs, which is higher than 110 TgC of VOCs from anthropogenic sources [7]. In recent years, many researchers have started assessing the total emissions of BVOCs in different regions. Chang et al. [8] estimated that the annual emission of BVOCs was 3.37 × 10^6^ gC·km^−2^ in the greater Hangzhou area. Pacheco et al. [9] estimated total isoprene emissions in Italy to be 6531.30 Gg in 2014. Although BVOCs have important functions in protecting plants from abiotic stresses [10], emissions of these BVOCs, especially in urban areas, can lead to secondary aerosols which contribute to particulate matter (PM_2.5_) and ground-level ozone pollution that threaten human health [11]. Therefore, we need to select suitable tree species and adjust the collocation of urban greening, which is a crucial solution to control BVOC pollution.

Isoprene is an important active substance among BVOCs, accounting for 44% of BVOCs [6]. Researchers have found that isoprene emissions from one single plant species are related to photosynthetic related factors [12,13]. Brilli et al. [14] found that isoprene emission is related to photosynthesis, stomatal conductance, and mesophyll conductance. Huve et al. [15] found that plant-derived methanol is closely related to changes in plant stomatal conductance. This may be affected by the isoprene synthases (IspS) and the supply of dimethylallyl diphosphate (DMADP) [16] and adenosine triphosphate (ATP) [17]. Thus, we speculate that the ability to emit isoprene is affected by photosynthesis, as evidenced by its relationship with the photosynthetic rate, stomatal conductance, and transpiration rate.

Previous studies mainly emphasized one single species and its influential factors, and oak was normally selected as the study plant [18]. However, there are great differences in isoprene emission rate among different species. For example, studies have found that *Populus*, *Quercus*, and *Ficus* are high emitters, while *Acer*, *Ulmus*, and *Juniperus* are considered as low-emission tree species [19,20,21,22]. On the one hand, the emission rate of common tree species in the subtropical monsoon area is rarely measured, so there is less reference for the selection of urban greening tree species. On the other hand, it is not clear whether photosynthesis is still the primary factor related to the interspecific difference.

In this paper, 10 common trees in subtropical metropolises were selected, and their isoprene emission rate in four seasons were measured to establish an emission inventory. The photosynthetic related parameters including photosynthetic rate, stomatal conductance, intercellular CO_2_ concentration and transpiration rate were also measured. Correlation analysis and principal component analysis (PCA) were used to determine the relationship and to test if these parameters can be used to predict isoprene emissions. According to emission inventory and main influencing factors, this paper can be helpful to the selection of urban greening tree species and targeted isoprene prevention, and can help the control of urban biogenic volatile organic compounds pollution.

## 2. Materials and Methods

### 2.1. Sampling Sites and Tree Species

**Figure 1:**
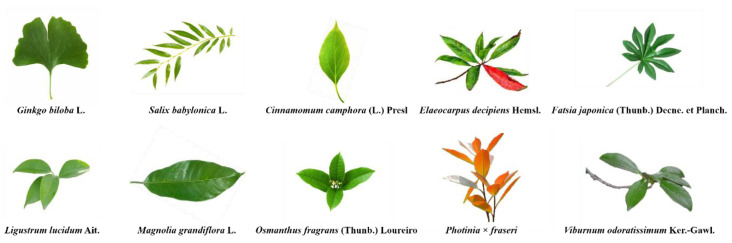
Leaves and shoots of the 10 tree species included in the study.

The sampling site was the botanical garden in Shanghai Jiao Tong University, which is located in the estuary of the Yangtze River (121°30′ E, 31°17′ N). Shanghai has a typical subtropical monsoon climate with distinct seasons and moderate precipitation throughout the year (approximately 1098 mm) [23]. The flood season is from May through September. The soil pH is 8.0–9.0, and the content of organic matter is at an intermediate level [24]. Evergreen broad-leaf tree species are typical plants in the subtropics. Thus, we selected 10 broad-leaf tree species (shown in Figure 1) as samples which are cultivated and grow well in the garden.

### 2.2. Measurement of Isoprene Emission Rate

In this experiment, we used the method described in Xiong et al. [25]; the enclosure sampling method was used to collect the gas emitted by trees, and then the time of flight mass spectrometry (TOF-MS) was used to measure and analyze BVOCs. The experiment was conducted four times, once each in June 2017, October 2017, January 2018, and April 2018 to represent summer, autumn, winter, and spring, respectively. Three adjacent trees of each tree species were selected and the results were averaged.

**Figure 2:**
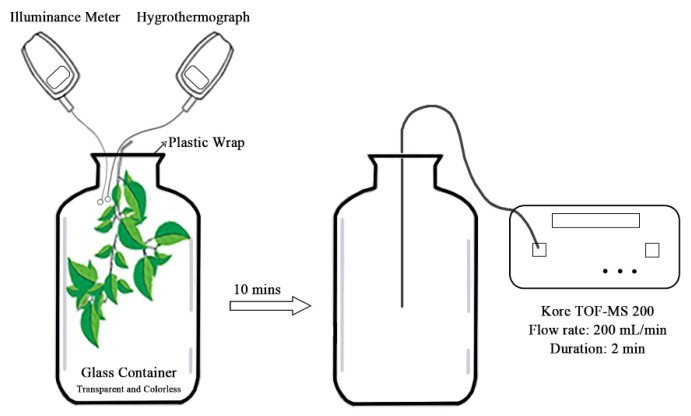
Sampling and measurement of isoprene emission rate. TOF-MS = time of flight mass spectrometry.

Sampling was conducted using a static enclosure system (Figure 2), which differs from the traditional system [21,26]. Teflon bags and absorbing tubes from the traditional approach were replaced with glass containers with 15 L volume. First, the gas was pumped in the container next to the sampling tree species to make the air in the container consistent with environment. Then the twigs were wrapped from three adjacent trees separately with three containers simultaneously and the gas was collected for 10 min. The specific requirements of twigs were as follows: the twigs were located outside the crown and 2/3 of the height of the tree with mature leaves and the length extending into the container was about 20 cm. At the same time, the illuminance meter (MQ-200, Apogee Instruments, Logan, UT, USA) and hygrothermograph (DT8002, Kejian, Guangdong, China) were used to measure the photosynthetically active radiation (PAR) and temperature around the experimental trees for several times and blank samples of the atmospheric environment around the tree species were also collected. After 10 min, the container was sealed and physiological parameters were measured with the leaves on these twigs (see Section 2.4 for details). Finally, the leaves were taken back to measure their biomass.

Then we chose time-of-flight mass spectrometry (TOF-MS) to measure BVOCs, which has been applied worldwide for gas analysis. Christof et al. [27] used this technology to determine acetaldehyde and the VOCs emissions of different wood cores were measured by Taiti et al. [28]. Since many studies have chosen to use gas chromatography and mass spectroscopy (GC-MS) for isoprene measurement in the past [21,29,30], before the formal experiment, we measured the emission rates of five species by both GC-MS and TOF-MS. The results are shown in Supplementary Materials Table S1. According to the independent samples t test, the smallest *p* value of the five trees is 0.148, which indicates that there is no significant difference (*p* > 0.05) between the results given by the two methods. Moreover, TOF-MS is more time-saving and can be observed in situ. Thus, we used the TOF-MS Analyzer (TOF-MS-200, Kore, Ely, UK) to measure emission rate. The samples were pumped into a membrane sampling system. Subsequently they entered the high-vacuum photoionization ion source through a special synthetic membrane, and the molecules to be tested were charged. The sample then entered the mass analysis chamber with a higher vacuum to analyze the volume concentration of the gas.

The amount of isoprene released from plants within 10 min can be obtained by subtracting the mass spectra of the blank base sample from the spectra of the analysis sample with TOF-MS-200 Analyzer software.

The isoprene emission rate calculation formula is as follows (Equation (1)):

(1) $$v=15\cdot c\cdot m^{-1}\cdot t^{-1}$$

where *v* is the emission rate of isoprene under environmental conditions (μg·g^−1^·h^−1^); *c* is the concentration of isoprene collected in the container; *m* is the dry weight of leaves (dried at 80 °C for 24 h after sampling and measurement of physiological parameters); 15 is the volume of the container (L), and *t* is the collection time 1/6 h.

### 2.3. Standardization of the Isoprene Emission Rate

Illumination and temperature have a remarkable influence on isoprene emission. Because of the variation of environmental conditions during sampling, all emission rates were standardized to 303 K and 1000 μmol·m^−2^·s^−1^ (Equations (2)–(4)) [31].

(2) $$I=\;I_{S}\times C_{L}\times C_{T}$$

where *I* is the emission rate at temperature *T* and PAR flux *L*, and *Is* is the emission rate under standard conditions (temperature = 303 K; PAR = 1000 μmol·m^−2^·s^−1^). *C_L_* is the light correction factor, and *C_T_* is the leaf temperature correction factor:

(3) $$C_{L}=\frac{\alpha C_{L_{1}}L}{{\left(1\;+\;\alpha^{2}L^{2}\right)}^{\frac{1}{2}}}$$

where α and *C_L1_* are empirical constants (α = 0.0027; *C_L_*_1_ = 1.066).

(4) $$C_{T}=\frac{exp\left[\frac{C_{T_{1}}\left(T-T_{S}\right)}{RT_{S}T}\right]}{1+exp\left[C_{T_{2}}\left(T-T_{M}\right)/RT_{S}T\right]}$$

where *R* is the ideal gas constant (8.314 J·K^−1^·mol^−1^), *T* is the leaf temperature, *C_T_*_1_ and *C_T_*_2_ are empirical constants (*C_T_*_1_ = 95,000 J·mol^−1^; *C_T_*_2_ = 230,000 J·mol^−1^), *T_M_* = 314 K, and *T_S_* is the standard temperature (303 K).

This formula is the most widely used one in isoprene emission rate standardization.

### 2.4. Measurement of Plant Photosynthesis and Related Physiological Parameters

Leaf photosynthesis and related physiological parameters (photosynthetic rate, stomatal conductance, transpiration rate, and intercellular CO_2_ concentration) of the 10 plant species in spring and summer were estimated using a LI-6400XT (LI-COR, Lincoln, NE, USA) portable photosynthetic instrument.

After sampling for isoprene emission, 10 leaves were selected from each twig for parameters determination in situ. The experiment was carried out at the best time of photosynthesis from 9 a.m. to 11 a.m. The abovementioned parameters were measured under natural condition (humidity of 30–50% and temperature of 22–27 °C in spring, 40–70% and 25–30 °C in summer). During the measurement of each leaf, data were recorded several times after the indication was stable, and the average value was taken as a repetition. The accurate temperature, relative humidity and PAR were simultaneously measured and recorded by the hygrothermograph and the illuminance meter.

### 2.5. Data Processing

Two-way ANOVA and Duncan’s multiple comparison were used to compare the isoprene emission rates between the tree species and to test the interaction between species and season. Pearson’s correlation was used to analyze the relationship between emission rate and photosynthesis and related parameters. PCA was conducted to obtain the dominant factors that can affect emission rate. Data processing mentioned above was conducted using SPSS (version 25, IBM, Chicago, IL, USA), Excel (version 16.32, Microsoft, Redmond, WA, USA) and PerformanceAnalytics, ggplot2, vegan, reshape2, tidyverse packages in RStudio (version 1.2.1335, RStudio, Boston, MA, USA).

## 3. Results

### 3.1. Isoprene Emission Rate of Different Trees in Four Seasons

**Figure 3:**
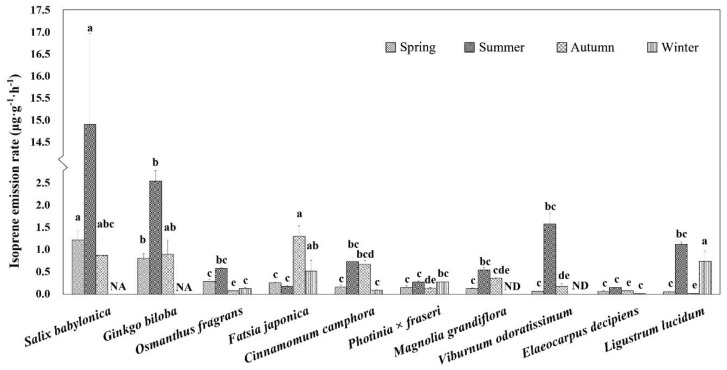
Isoprene emission rates of 10 typical plants in different seasons. a, b, c, d, e—within a season, species not sharing a common letter differ significantly (α = 0.01) in emission rate. ND = not detectable. NA = not available (*Salix babylonica* and *Ginkgo biloba* defoliated in autumn and winter).

The isoprene emission rates of 10 tree species in different seasons are as shown in Figure 3. According to the results of two-way ANOVA (see Supplementary Materials Table S2), the significant differences in isoprene emission rates existed not only between tree species but also seasons. Meanwhile, there were interaction between them, which may be due to different phenological characteristics among tree species.

In spring, the plant with the highest isoprene emission rate in Shanghai was *Salix babylonica* (1.22 μg g^−1^ h^−1^) The others were all less than 1 μg·g^−1^·h^−1^. *Viburnum odoratissimum* and *Elaeocarpus decipiens* emitted less than 0.1 μg·g^−1^·h^−1^. The lowest emission rate among all species was that of *Ligustrum lucidum* (0.05 μg·g^−1^·h^−1^). In summer, the emission rate of *S. babylonica* remained highest (14.94 μg·g^−1^·h^−1^) among all species. The emission rates of most species were less than 1 μg·g^−1^·h^−1^. In autumn, the emission rate of *Fatsia japonica* was the highest among the sample trees (1.3 μg·g^−1^·h^−1^). *E. decipiens* and *L. lucidum* remained at low levels, whereas *S. babylonica* emissions remained high. In winter, the species with the highest emission rate was *L. lucidum* (0.74 μg·g^−1^·h^−1^). Generally, the emission rates for all tree species were at a low level; the rates were less than 1 μg·g^−1^·h^−1^. *S. babylonica* and *Ginkgo biloba* are deciduous species, therefore, they were excluded from the comparison. The isoprene emissions of *M. grandiflora* and *V. odoratissimum* were undetected.

According to a comparison of the four seasons, the emission rate in summer was considerably higher than that in the other three seasons. The annual emission rate of *S. babylonica* (5.68 μg·g^−1^·h^−1^) was at a high level, whereas those of both *E. decipiens* (0.08 μg·g^−1^·h^−1^) and *Photinia* *× fraseri* (0.21 μg·g^−1^·h^−1^) were at a low level.

### 3.2. Photosynthesis and Related Physiological Parameters of Different Plants in Growing Seasons

The photosynthesis and related physiological parameters of 10 tree species are shown in Table 1.

During the growing seasons (spring to summer), the photosynthetic rate presented an uptrend; *S. babylonica* had the highest photosynthetic rate among the species in spring, whereas *L. lucidum* had the lowest. *Osmanthus fragrans* had the highest photosynthetic rate in summer, but the photosynthetic rate of *S. babylonica* also increased; the photosynthetic rate of *F. japonica* remained low.

The intercellular CO_2_ concentration of most tree species decreased from spring to summer. In spring *Cinnamomum camphora* and *Ginkgo biloba* exhibited the highest and lowest concentration, respectively. The species with the lowest concentration in summer was *C. camphora*, the species with the highest concentration was *F. japonica*.

The stomatal conductance of most plants increased remarkably from spring to summer. In spring, *S. babylonica* had the highest stomatal conductance and *O. fragrans* had the lowest. In summer *V. odoratissimum* and *P. fraseri* had the highest and lowest stomatal conductance. This may be due to the change of the opening or closing state of the stomatal pores affected by the temperature.

The transpiration rate showed a general uptrend from spring to summer. In spring, the highest transpiration rate was that of *S. babylonica*, whereas the lowest one was that of *P. fraseri*. In summer, the highest transpiration rate was that of *F. japonica*, and the lowest one was that of *E. decipiens*. The transpiration rate is also influenced by temperature. Therefore, it increased from spring to summer.

Measurements of photosynthetic related physiological characteristics showed great differences among plant species. They also differed from spring to summer. How these characteristics affect the emission of isoprene needs further discussion.

### 3.3. Correlations between Plant Parameters and Isoprene Emission Rate

**Figure 4:**
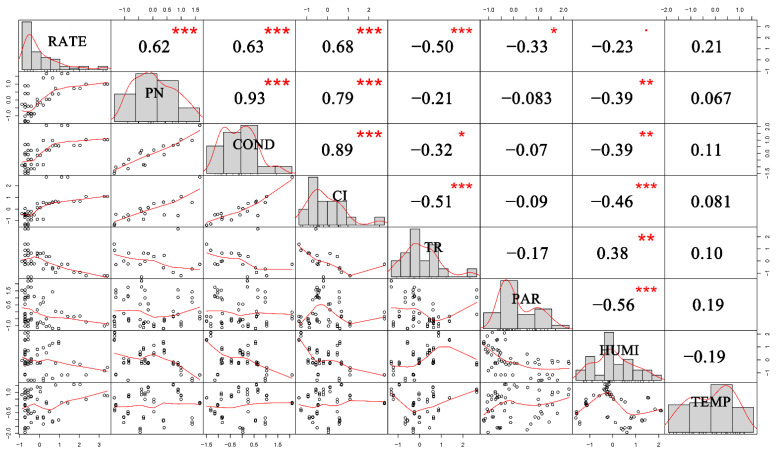
Correlation matrix of variables measured (*** *p* < 0.001; ** *p* < 0.01; * *p* < 0.05). The data has been standardized. RATE = isoprene emission rate; PN = net photosynthesis rate; COND = stomatal conductance; CI = intercellular CO_2_ concentration; TR = transpiration rate; PAR = photosynthetically active radiation; HUMI = humidity; TEMP = temperature.

In order to determine the correlation between factors and their impacts to the isoprene emission rate, Z-score normalization was employed to normalize the data of 10 tree species from spring and summer and a covariance matrix was deduced to directly reflect the relationships between variables as shown in Figure 4.

According to Figure 4, the isoprene emission rate (RATE) was strongly related to net photosynthesis rate (PN), stomatal conductance (COND), intercellular CO_2_ concentration (CI), transpiration rate (TR), and humidity (HUMI). Though net photosynthesis rate, stomatal conductance and intercellular CO_2_ concentration had a positive correlation with isoprene emission rate, transpiration rate and humidity were negatively related. In addition, net photosynthesis rate, stomatal conductance, intercellular CO_2_ concentration, and relative humidity were closely correlated with each other. However, photosynthetically active radiation (PAR) and temperature (TEMP) seemed to be unrelated to any other parameters, except that PAR was slightly negatively related to isoprene emission rate. Some of the factors were highly related, and dimensionality reduction should be applied here.

### 3.4. Key Factors of Isoprene Emission Rate Based on PCA

**Figure 5:**
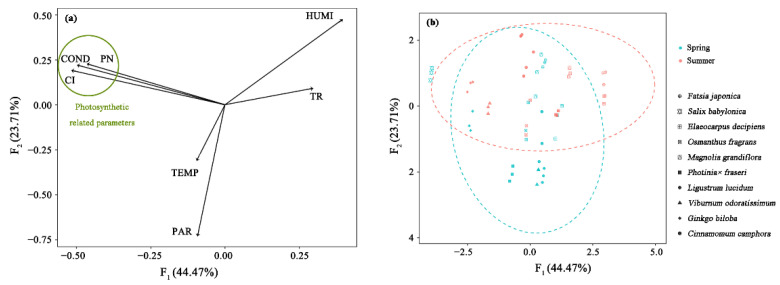
Principal component analysis of the 7 parameters. (**a**) Representation of the correlation coefficient between the principal components and the parameters. (**b**) Representation of the emission rate of 10 tree species in two seasons. F_1_ = the first principal component; F_2_ = the second principal component.

To comprehensively understand the main parameters influencing isoprene emission rate of 10 species, PCA was conducted to classify each factor and deduce the principal components. The results are as shown in Figure 5.

The analysis resulted in seven principal components (PCs), from which 68.18% of the total variance of the model was achieved with the first two PCs. The first component (F_1_) is the main indicator of the total variance of the data, which is responsible for 49.2% of the changes in all analyzed parameters. With regard to the relationship between the PCs and the parameters, F_1_ is negatively correlated to net photosynthesis rate, intercellular CO_2_ concentration, stomatal conductance, while positively correlated to humidity and transpiration rate (Figure 5a). The second factor (F_2_) is less important since it explains only 23.71% of the parameters. And it is mostly influenced by PAR, although it shows some relationship with humidity and temperature.

## 4. Discussion

### 4.1. Close Relationship between Photosynthesis Related Parameters and Isoprene Emission

As analyzed above, the first factor in PCA, “F_1_”, especially the net photosynthetic rate, is the most important contributor to the variance of isoprene emission rate. Although the mechanism of physiological process has not been studied in this paper, it is still proved that the differences of photosynthetic rate and isoprene emission are also interspecifically related. Moreover, some literatures have pointed out that photosynthetic activity is a significant variable affecting isoprene emission of individual plants [32,33,34,35,36,37]. Sanadze et al. [32] used isotope technology to prove that the substrate and energy of isoprene came entirely from photosynthesis. Monson et al. [33] also explained in detail how methylerythritol 4-phosphate (MEP) pathway in chloroplast constructed the relationship between photosynthesis and isoprene synthesis. The MEP pathway provides carbon substrates and then produces precursors for isoprene synthesis. Phosphoenolpyruvate (PEP) and glyceraldehyde 3-phosphate (G3P), which are produced through glycolysis or the C4 pathway, are transported to the chloroplasts, which leads to the formation of methyl erythritol after PEP and G3P undergo a complex process. Dimethylallyl pyrophosphate (DMAPP) is then formed and isomerized into isopentenyl pyrophosphate (IPP) [34], which are the activated forms of isoprene in plants. The MEP pathway also provides energy for the synthesis and release of isoprene. Photosynthesis can directly affect the release of ATP. Plants do not emit isoprene when leaves don’t photosynthesize [35]. 3-phosphoglyceric acid and 1,3-bisphosphoglyceric acid are possible candidates for the link between photosynthetic carbon metabolism and the regulation of isoprene emission, which is ATP dependent. All the points above suggest that the different photosynthetic abilities of different plants can affect their different isoprene emission capacities.

Furthermore, photosynthesis rate is under the influence of stomatal conductance as well as intercellular CO_2_ concentration. As the main substrate for photosynthesis, CO_2_ is acquired by stomata from the atmosphere. Stomatal conductance was found affecting the regulation of metabolites from photosynthesis [38]. Enhanced level of photosynthesis is related to the increase of stomatal conductance [39]. On the other hand, a low level of intercellular CO_2_ concentration provides conditions where photochemical energy colimits photosynthesis rate [40]. Also, high stomatal conductance is responsible for higher intercellular CO_2_ concentration, which has a positive impact on the electron flow of the carbon reduction pathway [41].

### 4.2. Other Factors Influencing Isoprene Emission

In addition to photosynthetic parameters, isoprene emission rate is also related to other factors. Humidity and transpiration rate were the last two factors to F_1_, although their effects were not so significant. Both humidity and transpiration rate show negative correlation with isoprene emission rate. The mechanism behind this was seldom discussed [42]. However, it is confirmed that relative humidity may change the pathways to form secondary organic aerosols from isoprene [43]. PAR and temperature are potential influential factors to photosynthesis activity and could further affect isoprene emission rate, which was long confirmed by previous studies [33,44]. However, the influence turns out to be small because, in order to obtain a standardized isoprene emission rate that is meaningful for cross-species comparison, most of the data were collected during “ideal” conditions where temperature and PAR were believed to be near the optimum.

### 4.3. Species Selection and Priority Control

Nowadays, much attention has been paid to the ecological service functions of plants, such as dust retention and noise reduction. However, in urban planning and construction, potential ecosystem disservices, such as promoting O_3_ generation through the emission of BVOC, must be considered. Some studies have found that deciduous trees account for a large proportion of urban greening [45], in which *Quercus*, *Salix*, *Populus*, and *Platanus* species generally have large isoprene emissions. Tree species with high emission have a higher share in Asia [46]. For example, in Shanghai, 47% of street trees are *Platanus acerifolia* [47]. In contrast, suburban and rural forests are usually dominated by evergreen trees [48], which mainly release monoterpenoids and have a slight impact on O_3_ formation. Therefore, in order to alleviate the adverse effects caused by plants, in urban greening planning, species with low isoprene emission, such as *E. decipiens* and *L. lucidum* according to the results in our study, should be given more attention, while tree species with high emission rates, such as *S. babylonica*, should be avoided as much as possible.

Meanwhile, we should also note that isoprene emissions are generally high in summer. This is basically consistent with the results of previous studies. Cai et al. [49] and Guo et al. [50] also indicated that VOC concentration increased in summer. Jardine et al. [30] found that the maximum emission rate of isoprene occurred at the highest light intensity. Therefore, people should pay attention to the prevention and control of BVOC in summer.

In addition to the emission inventory, more importantly, this study points out the possible common rules of high emission tree species. Although this study involves only a few species, the relationship between photosynthesis and isoprene emission was established by measuring the influencing factors among species, from which general patterns in BVOC emissions can be identified. For example, deciduous trees used to be considered as high isoprene emitters while evergreen trees as low emitters [51]. According to our results, tree species with high photosynthetic rate and low transpiration rate are more likely to be high isoprene emitters. Additionally, due to the great variability of vegetation emissions among individuals, and most researchers use their own instruments and unique sampling systems to measure, the results may not be directly comparable [21,52,53]. Therefore, in the future, it is more feasible to determine the emission level of BVOC through simple and uniform measurable photosynthetic parameters for tree species selection.

## 5. Conclusions

Generally, the isoprene emission rate of plants is the highest in summer and the lowest in winter. The main reason is the differences of the external environment and plants growing conditions among four seasons. The isoprene emission rate of *S. babylonica* always remains at a high level during the growing season, whereas that of *E. decipiens* and *L. lucidum* are at a low level throughout the year.

The differences in isoprene emission among different plant species was closely correlated with photosynthesis difference. The net photosynthetic related factors, including net photosynthesis rate, intercellular CO_2_ concentration, and stomatal conductivity, are the most significant factors summarized by the first principal component of a principal component analysis.

According to our study, considering the contribution of isoprene to O_3_ accumulation and PM production, especially in densely populated urban areas, the cultivation of low emission tree species such as *E. decipiens* and *L. lucidum* should be strengthened. Summer is the season of serious pollution, so more attention should be paid to the BVOCs control in this period. Although the tree species measured in this study were few, the possible commonness of high emission species is pointed out. Photosynthetic parameters can also be used as the basis of urban greening species selection to effectively alleviate urban air pollution.

## Figures and Tables

**Table 1:** Photosynthesis and related physiological parameters. Species are listed in decreasing order of photosynthetic rate in spring.

Species	Photosynthetic Rate (μmol CO_2_·m^−2^·s^−1^)		Intercellular CO_2_ Concentration (μmol CO_2_·mol^−1^)		Stomatal Conductance (mol H_2_O·m^−2^·s^−1^)		Transpiration Rate (mmol H_2_O·m^−2^·s^−1^)	
	Spr.	Sum.	Spr.	Sum.	Spr.	Sum.	Spr.	Sum.
*Salix babylonica*	9.6 ± 1.8	11.8 ± 2.2	273.3 ± 11.9	258.0 ± 13.5	0.14 ± 0.04	0.18 ± 0.05	3.98 ± 0.99	3.21 ± 0.73
*Ginkgo biloba*	9.2 ± 0.9	9.0 ± 1.5	260.7 ± 16.4	262.2 ± 11.8	0.11 ± 0.01	0.13 ± 0.01	2.49 ± 0.20	2.76 ± 0.19
*Cinnamomum camphora*	8.4 ± 1.1	10.6 ± 1.5	367.2 ± 13.5	216.6 ± 53.3	0.10 ± 0.01	0.09 ± 0.04	1.73 ± 0.08	1.66 ± 0.50
*Magnolia grandiflora*	7.6 ± 0.8	9.9 ± 1.9	296.8 ± 16.8	264.0 ± 6.5	0.09 ± 0.01	0.16 ± 0.01	1.76 ± 0.18	2.77 ± 0.30
*Elaeocarpus decipiens*	6.6 ± 0.8	12.3 ± 0.7	281.1 ± 14.4	226.9 ± 18.2	0.06 ± 0.02	0.08 ± 0.01	1.84 ± 0.33	1.30 ± 0.23
*Fatsia japonica*	6.4 ± 0.4	8.1 ± 0.7	239.6 ± 7.0	273.4 ± 13.2	0.09 ± 0.02	0.14 ± 0.02	1.98 ± 0.33	2.92 ± 0.36
*Viburnum odoratissimum*	6.3 ± 0.8	11.2 ± 0.8	272.5 ± 12.1	228.2 ± 7.0	0.09 ± 0.01	0.14 ± 0.02	2.14 ± 0.23	2.78 ± 0.26
*Osmanthus fragrans*	5.6 ± 0.6	14.0 ± 1.4	232.2 ± 32.5	251.2 ± 17.2	0.06 ± 0.01	0.12 ± 0.03	1.76 ± 0.26	2.05 ± 0.40
*Photinia* *× fraseri*	5.4 ± 0.7	10.3 ± 1.1	281.7 ± 15.5	218.9 ± 25.7	0.07 ± 0.01	0.07 ± 0.01	1.62 ± 0.26	1.82 ± 0.04
*Ligustrum lucidum*	4.9 ± 0.4	8.1 ± 0.6	272.2 ± 11.4	259.3 ± 16.8	0.06 ± 0.01	0.11 ± 0.03	1.80 ± 0.18	2.61 ± 0.60

## Data Availability

The data presented in this study are available in Supplementary Materials.

## Supplementary Materials

The following are available online at https://www.mdpi.com/1660-4601/18/3/954/s1, Table S1: Difference analysis of the two detection methods, Table S2: Results of Two-Way ANOVA of species and seasonal differences on emission rate.
