# Supplementary material for: Photosynthesis and Related Physiological Parameters Differences Affected the Isoprene Emission Rate among 10 Typical Tree Species in Subtropical Metropolises
Source: Int J Environ Res Public Health. 2021 Jan 22;18(3):954. doi: 10.3390/ijerph18030954 (PMC7908470; doi:10.3390/ijerph18030954)
Supplement: Supplementary file 1 [file ijerph-18-00954-s001.pdf]

International Journal of
Environmental Research and Public Health

Table S1. Difference analysis of the two detection methods

Species	Isoprene Emission Rate (μg·g^{-1}·h^{-1})		t	Sig.
	GC-MS method	TOF-MS method		
Salix babylonica	12.72 ± 1.03	14.94 ± 2.88	1.046	0.355
Ginkgo biloba	2.19 ± 0.23	2.54 ± 0.33	1.305	0.262
Cinnamomum camphora	0.84 ± 0.22	0.73 ± 0.08	-0.689	0.529
Ligustrum lucidum	1.58 ± 1.18	1.12 ± 0.14	-0.667	0.541
Viburnum odoratissimum	1.14 ± 0.12	1.58 ± 0.33	1.787	0.148

Table S2. Results of Two-Way ANOVA of species and seasonal differences on emission rate

	Df	SumsOfSqs	MeanSqs	F.Model	R^2	Pr(>F)	
Species	9	236.33	26.2585	66.503	0.354	0.001	***
Season	3	70.62	23.5413	59.621	0.10579	0.001	***
Species:Season	23	332.21	14.4438	36.581	0.49762	0.001	***
